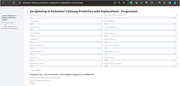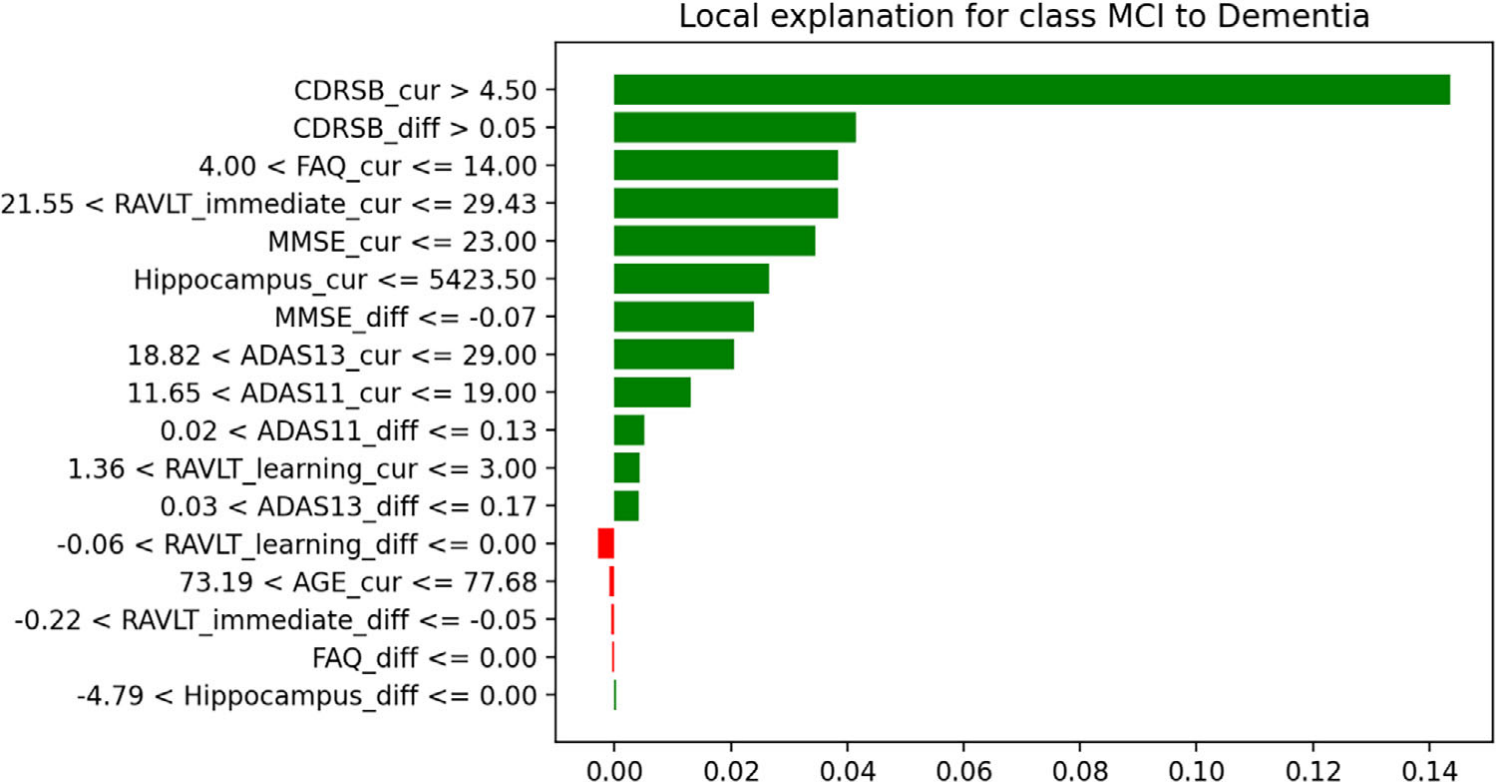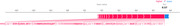# Web Application for Deciphering Alzheimer’s Disease Prediction and Progression with Explanations

**DOI:** 10.1002/alz.085960

**Published:** 2025-01-03

**Authors:** Ben George Ephrem, Abraham Varghese, Vinu Sherimon, Prasanth Gouda

**Affiliations:** ^1^ University of Technology and Applied Sciences, Muscat, Muscat Oman; ^2^ University of Technology and Applied Sciences, Muscat Oman; ^3^ National University, Muscat, Muscat Oman

## Abstract

**Background:**

Early detection and personalized care for Alzheimer’s Disease (AD) mitigate the devastating consequences for millions of people around the globe. In the current scenario, there is a lack of user‐friendly AI applications for predicting and understanding the progression of AD. The application should address the critical need for a predictive analytics tool that offers timely and transparent insights by utilizing the patient data. The research aims to develop an AI‐driven application for predicting and interpreting the disease progression.

**Method:**

The application utilizes ADNI dataset, where the available patient data has been oversampled to 13752 to balance the target class. The Random Forest Classifier, a powerful machine learning technique was used to train the set of selected features including age, cognitive scores from standardized tests, and brain imaging data. Based on these inputs, the model accurately predicts an individual’s current cognitive state, categorizing them as Normal, Mild Cognitive Impairment (MCI), or Dementia. The application also utilizes the patient’s historical and current data to predict the progression from MCI to Dementia, or Normal to MCI, or remaining in the current cognitive state. The application uses LIME (Local Interpretable Model‐agnostic Explanations) and SHAP (SHapley Additive exPlanations) for transparent explanations and providing clear insights into the rationale behind interpretability in predicting each class using different visualizations.

**Result:**

The application (https://alzheimer‐disease‐prediction‐progression‐explanations.streamlit.app/) demonstrates accuracy in both initial diagnosis and progression prediction (Figure 1). Explainable AI techniques provide user‐friendly insights into model reasoning, promoting trust and understanding (Figure 2 and 3). The user‐friendly interface facilitates data input and interpretation for both healthcare professionals and individuals.

**Conclusion:**

The AI‐driven application emerges as a valuable tool in AD prediction and analysis. The transparent and interpretable nature of the model, facilitated by LIME and SHAP, enhances trust and understanding. Early detection and progression prediction capabilities allow healthcare professionals with timely interventions to care the patients.